# Cytotoxic Activities against Breast Cancer Cells of Local *Justicia gendarussa* Crude Extracts

**DOI:** 10.1155/2014/732980

**Published:** 2014-12-11

**Authors:** Zahidah Ayob, Siti Pauliena Mohd Bohari, Azman Abd Samad, Shajarahtunnur Jamil

**Affiliations:** ^1^Department of Biotechnology and Medical Engineering, Faculty of Biosciences and Medical Engineering, Universiti Teknologi Malaysia (UTM), 81310 Johor Bahru, Malaysia; ^2^Department of Chemistry, Faculty of Science, Universiti Teknologi Malaysia (UTM), 81310 Johor Bahru, Malaysia

## Abstract

*Justicia gendarussa* methanolic leaf extracts from five different locations in the Southern region of Peninsular Malaysia and two flavonoids, kaempferol and naringenin, were tested for cytotoxic activity. Kaempferol and naringenin were two flavonoids detected in leaf extracts using gas chromatography-flame ionization detection (GC-FID). The results indicated that highest concentrations of kaempferol and naringenin were detected in leaves extracted from Mersing with 1591.80 mg/kg and 444.35 mg/kg, respectively. Positive correlations were observed between kaempferol and naringenin concentrations in all leaf extracts analysed with the Pearson method. The effects of kaempferol and naringenin from leaf extracts were examined on breast cancer cell lines (MDA-MB-231 and MDA-MB-468) using MTT assay. Leaf extract from Mersing showed high cytotoxicity against MDA-MB-468 and MDA-MB-231 with IC_50_ values of 23 *μ*g/mL and 40 *μ*g/mL, respectively, compared to other leaf extracts. Kaempferol possessed high cytotoxicity against MDA-MB-468 and MDA-MB-231 with IC_50_ values of 23 *μ*g/mL and 34 *μ*g/mL, respectively. These findings suggest that the presence of kaempferol in Mersing leaf extract contributed to high cytotoxicity of both MDA-MB-231 and MDA-MB-468 cancer cell lines.

## 1. Introduction

Breast cancer is the second largest cancer after lung cancer in the world and the most common malignancy among women [1]. In Malaysia, the most frequent cancers are breast cancer (18.1%), colorectal cancer (12.3%), and lung cancer (10.2%); these three cancers affect both women and men [2]. Currently, the most common approaches for treating human breast cancer include surgery, radiotherapy, hyperthermia, hormone therapy, and chemotherapy [3].

Breast cancers can be classified by stage, pathology, grade, and expression of oestrogen receptor (ER), progesterone receptor (PR), or human epidermal growth factor receptor (Her2/neu) [4]. The two types of breast cancer cells that have gained interest among investigators and medical research laboratories are MDA-MB-231 and MDA-MB-468. MDA-MB-231 cells are characterised as ER-, PR-, and Her2/neu-negative/basal-B mammary carcinoma, while MDA-MB-468 cells are characterised as ER-, PR-, and Her2/neu-negative/basal-A mammary carcinoma [4]. MDA-MB-231 and MDA-MB-468 cells were derived from the pleural effusions of 51-year-old female patients. MDA-MB-231 cells were derived from a Caucasian female, while MDA-MB-468 cells were derived from an African American female [5–7].

There is strong social interest in natural remedies, and more than 80% of the world population considers traditional medicine as their source of primary health care [8]. Indeed, there has been a worldwide effort to discover new anticancer agents from medicinal plants, and various experimental models of natural products have resulted in anticancer agents [9, 10].

One of the potential medicinal plants that is being investigated in our laboratory is* J. gendarussa*, which is also known by its common name Gendarussa. This plant is a member of the Acanthaceae family that can be found ubiquitously in many countries, including Indonesia, Sri Lanka, India, and Malaysia [11]. The roots and leaf extracts of* J. gendarussa *have been demonstrated to treat chronic rheumatism, inflammation, bronchitis, headache, arthritis, vaginal discharges, dyspepsia, eye disease, and fever [12].

Previous reports demonstrated that* J. gendarussa *leaf extracts have been used traditionally as a male contraceptive agent by several ethnic groups in the central part of Papua, Indonesia. This extract is able to inhibit mouse spermatozoa penetration of mice ovum [13].* J. gendarussa *methanolic leaves and root extracts showed cytotoxic activity against brine shrimp in the brine shrimp lethality assay with IC_50_ values of 48.71 *μ*g/mL and 93.25 *μ*g/mL, respectively [14]. In addition,* J. gendarussa* leaves and stem extracts were reported to have anticancer, antioxidant, antibacterial, antifungal, antiangiogenic, anthelmintic, and hepatoprotective activities [15–23].

Phytochemical studies on leaves from* J. gendarussa *revealed the presence of flavonoids, alkaloids, triterpenoidal saponins, amino acids, aromatic amines, stigmasterol, and lupeol [18, 24–27]. Our previous study on green callus and* in vitro *leaf extracts of* J. gendarussa* detected two flavonoids, that is, kaempferol and naringenin using GC-FID [28]. Both flavonoids were also detected in the methanolic leaf extract of* J. gendarussa* using the same method [29]. Bioactivity studies on both flavonoids found that it exhibited strong antioxidant and inhibitory effects on cholesterol in HepG2 cancer cells [30–32]. Kaempferol also inhibited pancreatic cancer cell (MIAPaCa-2 and Panc-1) proliferation, induced cancer cell apoptosis, and prevented arteriosclerosis [30, 33]. Naringenin demonstrated cytotoxic effects against breast cancer cells (MCF-7) and suppressed apoptosis in mouse leukaemia P388 cells [34–36]. Our previous study on both flavonoids showed strong cytotoxic activity against colonic (HT-29), cervical (HeLa), and pancreatic (BxPC-3) cells [29].

To the best of our knowledge, this is the first study of the effects of* J. gendarussa *leaf extracts against human breast cancer cell lines (MDA-MB-231 and MDA-MB-468). This study was performed to screen the cytotoxic activities of methanolic leaf extracts from five different locations (Mersing, Muar, Skudai, Batu Pahat, and Pulai) in Johor and two flavonoids (naringenin and kaempferol) against breast cancer cell lines. The quantification of kaempferol and naringenin content in leaf extracts of* J. gendarussa* using GC-FID was also carried out.

## 2. Methods and Materials

### 2.1. Plant Materials

*J. gendarussa* plants were collected from five different locations in Johor (Mersing, Muar, Skudai, Batu Pahat, and Pulai) and maintained in a greenhouse at the Faculty of Biosciences and Medical Engineering, Universiti Teknologi Malaysia (UTM). The* J. gendarussa* plant was identified by Dr. Richard Chung Cheng Kong, senior research officer of the Forest Research Institute of Malaysia (FRIM). The voucher specimen (PID-100214-06) was deposited at Herbarium Management Branch, Flora Biodiversity Program, Forest Biodiversity Division, FRIM, Kepong, Selangor, Malaysia.

### 2.2. General Chemicals

Commercial standards (kaempferol and naringenin) were purchased from Sigma-Aldrich (Subang Jaya, Selangor, Malaysia). Tamoxifen was used as a positive control in the MTT assay. All samples were diluted with 0.1% of dimethylsulfoxide (DMSO), which has no effect on cell viability [37].

### 2.3. Preparation of Extracts

The* J. gendarussa *leaves were air-dried for 4 weeks. The dried leaves were ground into small particles and approximately 50 g of small particles was soaked into 1000 mL of methanol at room temperature for 72 hours in a ratio of 1 : 20 (w/v) [10]. The mixtures were filtered through sterile cotton and filtered again using Whatman number 1 filter paper to obtain methanolic supernatants. The filtered methanolic extract was evaporated at 40°C under reduced pressure by using an EYELA N-1000 rotary evaporator (Bohemia, NY, USA). The dried crude extract was kept at 4°C prior to use.

### 2.4. Quantification of Flavonoids in Leaf Extracts

GC-FID and quantitative analysis were performed according to previously published method [38]. GC-FID (HP-6890N, Agilent, USA) equipped with a HP-5 fused silica capillary column (30.0 m × 0.32 mm ID × 0.25 *μ*m) was used. The temperature programmed was 100°C held for 1 minute and then ramped to 275°C at 10°C/min and held for 17 minutes at 275°C. The injection temperature was 275°C. The flow rate of the carrier gas (helium) was 1 mL/min. A split ratio of 50 : 1 was used. A quantity of 5 *μ*L of leaf extract and standards was injected. The chromatographic data were recorded and processed using Agilent Cerity QA-QC software.

### 2.5. Cell Culture

MDA-MB-231 (basal-B mammary carcinoma) and MDA-MB-468 (basal-A mammary carcinoma) breast cancer cell lines and CHO (Chinese hamster ovary) normal cell line were obtained from American Type Culture Collection (ATCC) and as a generous gift from Dr. Salehhuddin Hamdan (Animal Cell Culture Laboratory, Faculty of Biosciences and Medical Engineering, UTM). MDA-MB-231 and MDA-MB-468 breast cancer cell lines were cultured in Dulbecco's modified Eagle's medium (DMEM), while CHO normal cells were cultured in Roswell Park Memorial Institute 1640 (RPMI-1640) medium supplemented with 10% v/v foetal bovine serum (FBS), 100 U/mL of penicillin, and 100 *μ*g/mL of streptomycin as a complete growth medium. Cells were maintained in 25 cm^2^ flasks and incubated in a humidified incubator (CO_2_ Water-Jacketed Incubator NuAire, Fernbrook Lane, Plymouth, USA) at 37°C with 5% CO_2_. All materials were obtained from Gibco (Gibco, Bio-Diagnostics, Petaling Jaya, Selangor, Malaysia).

### 2.6. MTT Assay

Cytotoxicity testing was performed using 3-(4,5-dimethylthiazol-2-yl)-2,5-diphenyltetrazolium bromide (MTT, Sigma) according to the method reported in previous studies [29, 39]. In this assay, cells were harvested after reaching 80% confluence. Before starting the MTT assay, cells were optimised at different seeding densities ranging from 2.0 × 10^3^ cell/mL to 1.0 × 10^6^ cell/mL in light to determine appropriate seeding number for the experiment. Each well of the microtiter plate (96-well) was filled with 100 *μ*L of cell suspension (MDA-MB-231, MDA-MB-468, and CHO with the seeding number; 5 × 10^4^ cell/mL) in complete growth medium. After 24 hours of incubation, cells were treated with leaf extracts of different concentrations ranging from 7.81 to 1000 *μ*g/mL, with a total well volume of 200 *μ*L with technical replicates. Microtiter plates were further incubated for 72 hours with plant extracts. After 72 hours of incubation, 20 *μ*L of MTT (a stock solution of 5 mg/mL in PBS) was added to each well, and the plates incubated for 4 hours at 37°C. Medium from each well was carefully removed without disturbing the MTT crystals in wells. The MTT formazan crystals were dissolved by the addition of 1 M HCl and 100 mM isopropanol to each well. After solubilising the purple formazan, absorbance was measured using a BioRad microplate reader (Shinagawa-ku, Tokyo, Japan) at a wavelength of 575 nm. Cytotoxic activity was recorded as IC_50_, which is the concentration necessary to reduce the absorbance of treated cells by 50% compared to the control (untreated cells) [40].

### 2.7. Statistical Analysis

All samples were run in three replicates. Data obtained were analysed using SPSS software for Windows (SPSS 16.0 for Windows Evaluation Version software, SPSS Inc., USA). The normality of the data was tested using the Shapiro-Wilk test. The data were analysed using the Independence *t*-test for normal data and Mann-Whitney *U* test for nonnormal data. The correlations were analysed using the Pearson correlation test [41]. Differences were considered to achieve significance for probability *P* < 0.05.

## 3. Results

**Figure 1:**
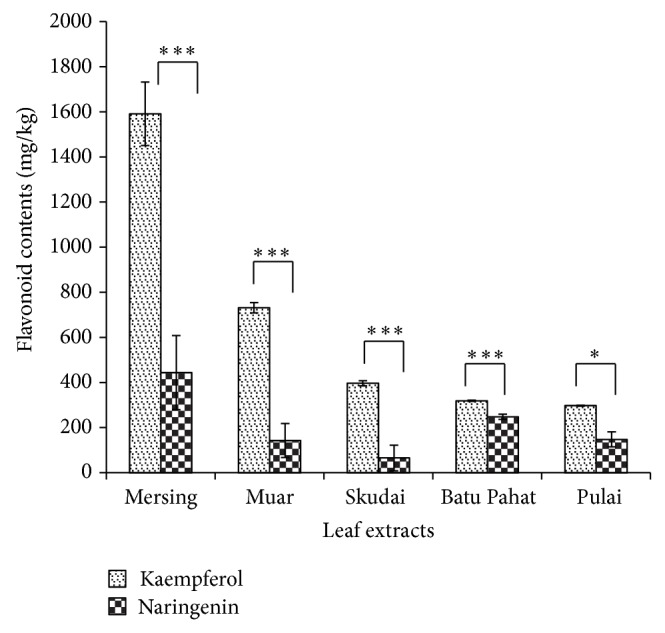
Distribution of kaempferol and naringenin contents in leaf extracts from five different locations. Each result is the mean of 3 replicates. Error bars represent standard deviations (STDEV). Results that are significantly different ^*^ *P* < 0.05, ^**^ *P* < 0.01, and ^***^ *P* < 0.001 are marked with an asterisk.

Phytochemical analysis of* J. gendarussa *leaf extracts showed that kaempferol and naringenin were quantified from five different locations by GC-FID.  Figure 1 shows the distribution of kaempferol and naringenin contents in leaf extracts.

In this study, cytotoxicity of* J. gendarussa *leaf extracts from five different locations and flavonoids (kaempferol, naringenin, and a mixture of kaempferol and naringenin) were tested against breast cancer cell lines (MDA-MB-231 and MDA-MB-468) and a normal cell line (CHO) using MTT assay. Tamoxifen was used as a positive control. The IC_50_ values obtained referred to 50% of cells inhibited by plant extracts [42]. In a previous study, cytotoxicity was evaluated based on IC_50_ values, where IC_50_ values below 20 *μ*g/mL were considered cytotoxic, from 21 to 40 *μ*g/mL were considered weak cytotoxic, and above 40 *μ*g/mL were not considered cytotoxic [40, 43, 44].

Table 1 represents the IC_50_ values of* J. gendarussa *leaf extracts, flavonoids, and tamoxifen. Overall, tamoxifen showed cytotoxic activity against CHO and MDA-MB-231 cells with IC_50_ values of 8 *μ*g/mL and 12 *μ*g/mL, respectively, compared to MDA-MB-468 cell with IC_50_ values of 27 *μ*g/mL.

**Figure 2:**
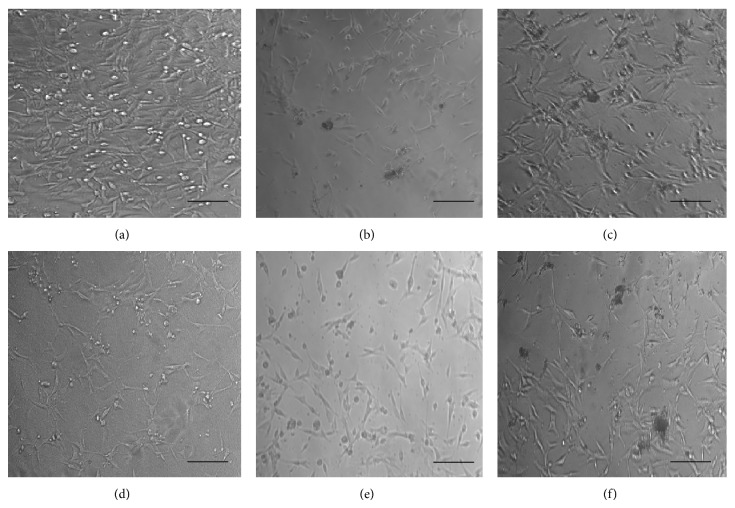
Morphology changes of MDA-MB-231 cells when treated with leaf extracts. (a) MDA-MB-231 cells without any treatment; (b) leaf extract from Mersing (IC_50_: 40 *μ*g/mL); (c) leaf extract from Muar (IC_50_: 275 *μ*g/mL); (d) leaf extract from Skudai (IC_50_: 61 *μ*g/mL); (e) leaf extract from Batu Pahat (IC_50_: 538 *μ*g/mL); and (f) leaf extract from Pulai (IC_50_: 250 *μ*g/mL). Scale bars: 100 *μ*M.

Morphological changes of cells were observed under an inverted fluorescence microscope (Nikon ECLIPSE T*i*-S, Shinagawa-ku, Tokyo, Japan) (100x magnification) after 72 hours of treatment. The methanolic leaf extracts from various locations were used to treat MDA-MB-231 cancer cell lines and revealed morphology changes (Figures 2(b), 2(c), 2(d), 2(e), and 2(f)) compared to nontreated cells (Figure 2(a)).

**Figure 3:**
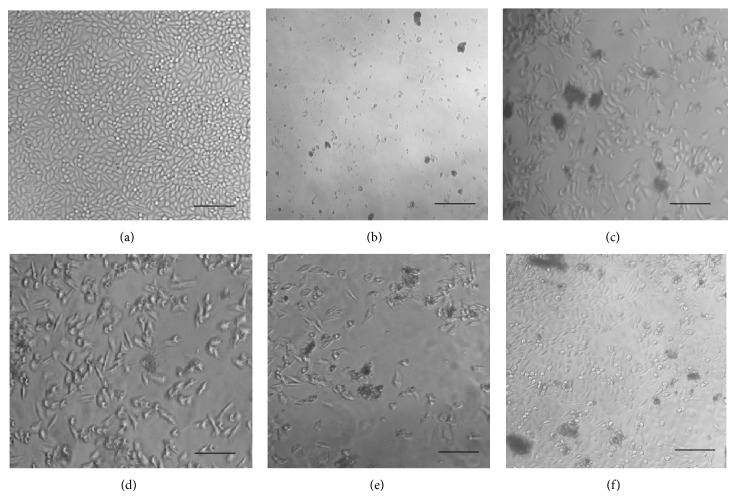
Morphological changes of MDA-MB-468 cells when treated with leaf extracts. (a) MDA-MB-468 cells without any treatment; (b) leaf extract from Mersing (IC_50_: 23 *μ*g/mL); (c) leaf extract from Muar (IC_50_: 160 *μ*g/mL); (d) leaf extract from Skudai (IC_50_: 259 *μ*g/mL); (e) leaf extract from Batu Pahat (IC_50_: 398 *μ*g/mL); and (f) leaf extract from Pulai (IC_50_: 299 *μ*g/mL). Scale bars: 100 *μ*M.

Morphological changes were revealed after methanolic leaf extract treatment of MDA-MB-468 cancer cell lines (Figures 3(b), 3(c), 3(d), 3(e), and 3(f)) compared to nontreated cells (Figure 3(a)).

**Figure 4:**
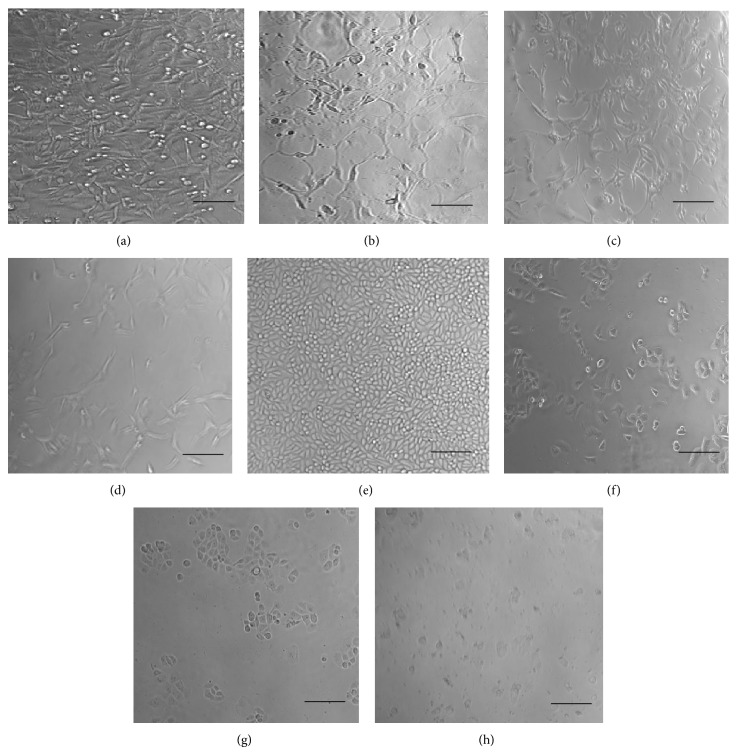
Morphology of MDA-MB231 and MDA-MB-468 cells when treated with kaempferol, naringenin and a mixture of kaempferol and naringenin. (a) MDA-MB-231 cells without any treatment, control; (b) MDA-MB-231 cells treated with kaempferol (IC_50_: 34 *μ*g/mL); (c) MDA-MB-231 cells treated with naringenin (IC_50_: 238 *μ*g/mL); (d) MDA-MB-231 cells treated with a mixture of kaempferol and naringenin (IC_50_: 43 *μ*g/mL); (e) MDA-MB-468 cells without any treatment, control; (f) MDA-MB-468 cells treated with kaempferol (IC_50_: 23 *μ*g/mL); (g) MDA-MB-468 cells treated with naringenin (IC_50_: 70 *μ*g/mL); (h) MDA-MB-468 cells treated with a mixture of kaempferol and naringenin (IC_50_: 44 *μ*g/mL). Scale bars: 100 *μ*M.

The morphology changes of MDA-MB-231 (Figures 4(b), 4(c), and 4(d)) and MDA-MB-468 (Figures 4(f), 4(g), and 4(h)) cancer cell lines when treated with kaempferol, naringenin and a mixture of kaempferol and naringenin compared to nontreated MDA-MB-231 and MDA-MB-468 cancer cell lines (Figures 4(a) and 4(e)), respectively.

## 4. Discussion

Phytochemical analysis of kaempferol and naringenin in leaf extracts from five locations was evaluated and shown in Figure 1. The highest concentrations of kaempferol and naringenin were found in leaf extracts from Mersing with 1591.80 mg/kg and 444.35 mg/kg, respectively. Positive correlations were observed between kaempferol and naringenin in all leaf extracts when analysed using the Pearson method. In addition, there was a significant difference in the kaempferol and naringenin distribution contents of leaf extracts from five different locations.

The cytotoxicity profile of* J. gendarussa *leaf extracts from five different locations and flavonoids (kaempferol, naringenin, and a mixture of kaempferol and naringenin) against MDA-MD-231, MDA-MB-468, and CHO cells are shown in Table 1. The inhibitory effects of all leaf extracts against breast cancer cell lines were decreased in a dose dependent manner, and these trends are consistent with previous studies [10, 42, 45, 46]. The IC_50_ values of the leaf extract from Mersing (40 *μ*g/mL) showed weak cytotoxicity, followed by leaf extracts from Skudai (61 *μ*g/mL), Batu Pahat (250 *μ*g/mL), Muar (275 *μ*g/mL), and Pulai (275 *μ*g/mL) against MDA-MB-231 breast cancer cells. IC_50_ values of the leaf extract from Mersing (23 *μ*g/mL) showed weak cytotoxicity, followed by leaf extracts from Muar (160 *μ*g/mL), Skudai (259 *μ*g/mL), Batu Pahat (299 *μ*g/mL), and Pulai (398 *μ*g/mL) against MDA-MB-468 cell lines. The percent cell viability of leaf extracts and flavonoids was compared to the control (untreated cell). The results demonstrate that there was a significant difference in IC_50_ values of each leaf extract against MDA-MB-231 and MDA-MB-468 cell lines (Tables 2 and 3). Because both flavonoids were present in high concentrations in leaf extracts, it is suggested that cytotoxic effects were mainly due to the presence of these flavonoids in elucidating tumour suppressive effects.

Table 1 also shows the ability of kaempferol, naringenin, and a mixture of kaempferol and naringenin to inhibit the proliferation of breast cancer cell lines in this study. However, kaempferol showed weak cytotoxicity, with IC_50_ values of approximately 34 *μ*g/mL (MDA-MB-231) and 23 *μ*g/mL (MDA-MB-468). This was followed by naringenin, with IC_50_ values of approximately 238 *μ*g/mL (MDA-MB-231) and 70 *μ*g/mL (MDA-MB-468). The mixture of flavonoids also showed weak cytotoxicity, with IC_50_ values of approximately 43 *μ*g/mL (MDA-MB-231) and 44 *μ*g/mL (MDA-MB-468). It is proposed that kaempferol associated highest cytotoxicity against breast cancer cell lines compared to naringenin and a mixture of kaempferol and naringenin.  Table 4 shows that there was a significant difference between the control with MDA-MB-231 and MDA-MB-468 treated cells for IC_50_ values of flavonoids, except for kaempferol against MDA-MD-231. The leaf extracts and flavonoids also showed low cytotoxicity toward CHO cells (Table 1). This indicates a lack of selectivity in the cytotoxicity between cancer and normal cells by the leaf extracts and flavonoids [47].

However, the current study also has contradictory results. It is shown in Figures 1, 2, and 3 that treated cells showed more prominent growth inhibition and shrinkage of the cells when compared to untreated cells that remained confluent. Many factors may have influenced these contradictory results. The plant source, environmental and geographic conditions, cell lines, and seeding number used in this study were completely different from those used in published works [48–50]. Thus, the results presented in this study were not totally in agreement with published [40, 43, 44] statements of IC_50_ values ranging toward crude extracts. Moreover, different plant extracts exhibited different effects on the proliferation of cells according to properties of the compounds [48]. This was because selectivity could be due to the sensitivities of cell lines against the active compounds in crude extracts that have a specific response [51, 52]. Overall,* J. gendarussa* leaf extracts and flavonoids were considered to hold promising anticancer effects on MDA-MB-231 and MDA-MB-468 cells.

The results of* J. gendarussa* leaf extract from Mersing showed less of an effect against MDA-MB-231 compared to MDA-MB-468 (Table 1). This suggests that the effects of active compounds, particularly flavonoids, on MDA-MB-231 are less cytotoxic compared to those on MDA-MB-468 cell lines. MDA-MB-231 is an oestrogen receptor (ER-negative) cell line that contains more than one cell population and is highly aggressive, invasive, and poorly differentiated from human breast cancer cell lines [53, 54]. MDA-MB-468 cells were most resistant to hyperacetylation and DNA degradation by drug treatments. This suggests that the MDA-MB-468 cell line has a phenotypic difference from and is less invasive than MDA-MB-231 [4]. In a previous study,* T. crispa* and* M. calabura* methanolic leaf extracts produced IC_50_ values of approximately 52.5 *μ*g/mL and more than 100 *μ*g/mL, respectively [10, 42]. However,* J. gendarussa *leaf extract from Mersing showed an IC_50_ value of 40 *μ*g/mL, exhibiting higher toxicity compared to other leaf extracts. It is suggested that* J. gendarussa *leaf extract from Mersing has cytotoxicity potential against MDA-MB-231 cells compared to other plant leaf extracts.

Based on the collected data, kaempferol showed the highest cytotoxicity against MDA-MB-468, followed by MDA-MB-231 and naringenin. These results are consistent with other studies showing weak inhibition of naringenin by other flavonoids [55]. A previous study reported that flavonoids with hydroxyl substituents at the 4′ and 7 positions were invariably oestrogenic, and an additional hydroxyl group at the 5th position increased estrogenic activity [56]. The present study supports this claim [56]. Previous work also demonstrated that naringenin showed a stronger oestrogenicity when tested on BT-474 human breast cancer cell lines [57]. It is plausible to suggest that both flavonoids contribute strong oestrogenic potency to the inhibition of oestrogen-independent breast cancer cells, MDA-MB-231 and MDA-MB-468.

Table 1 also shows the cytotoxicity of* J. gendarussa *leaf extracts, flavonoids, and tamoxifen on a normal cell line (CHO). CHO cells were a positive control used for comparison with the cytotoxicity activity on MDA-MB-231 and MDA-MB-468 breast cancer cell lines. Comparisons of* J. gendarussa *leaf extracts, flavonoids, and tamoxifen were performed in terms of IC_50_ values between breast cancer and normal cell lines. Tamoxifen was demonstrated to be cytotoxic to CHO cell lines (IC_50_ < 20 *μ*g/mL) in this study. Although the IC_50_ values of leaf extracts and flavonoids were not as low as tamoxifen, they had low toxicity against CHO cells. Due to its high toxicity in CHO cells, the continuous use of tamoxifen can cause adverse side effects [58]. If these results also occur* in vivo*, these leaf extracts would be considered safe for human consumption and could be used for further toxicity and clinical studies. Hence, the use of leaf extracts and flavonoids as anticancer agents in combination with other therapeutic drugs may reduce the adverse effects of drugs. Therefore, more comprehensive studies involving animal and clinical investigations are required.

## 5. Conclusion

In conclusion,* J. gendarussa *leaf extract from Mersing and kaempferol were considered cytotoxic against MDA-MB-231 and MDA-MB-468 compared to other leaf extracts and naringenin. Leaf extract from Mersing showed high contents of kaempferol and naringenin compared to other leaf extracts when quantified using GC-FID. Our results suggest that there is a correlation between the presence of kaempferol in the leaf extract from Mersing with the level of cytotoxicity against both breast cancer cell lines. These data will be beneficial to other researchers and validate the potential use of* J. gendarussa* leaves as novel anticancer agents.

## Figures and Tables

**Table 1:** Comparison of IC_50_ values between *J. gendarussa* leaf extracts, flavonoids, and tamoxifen in breast cancer cell lines.

	IC_50_ values (*μ*g/mL)		
	MDA-MB-231	MDA-MB-468	CHO
Leaf extract			
Mersing	40	23	28
Muar	275	160	108
Skudai	61	259	88
Batu Pahat	538	398	190
Pulai	250	299	305
Compounds			
Kaempferol	34	23	14
Naringenin	238	70	21
Mixture of kaempferol and naringenin	43	44	NT
Tamoxifen	12	27	8

NT: not tested.

**Table 2:** Percentage viability of MDA-MB-231 cells in leaf extracts from five different locations.

Concentration (*μ*g/mL)	7.81	15.63	31.25	62.5	125	250	500	1000
Leaf extract from Mersing	60.25 ± 0.06^***^	54.35 ± 0.05^***^	55.95 ± 0.08^***^	33.91 ± 0.07^***^	24.08 ± 0.02^***^	23.78 ± 0.02^***^	17.60 ± 0.03^***^	29.85 ± 0.08^**^

Leaf extract from Muar	79.26 ± 0.07^*^	72.89 ± 0.07^*^	71.35 ± 0.08^*^	70.34 ± 0.07^*^	63.49 ± 0.09^*^	54.58 ± 0.04^**^	8.29 ± 0.01^***^	11.54 ± 0.02^***^

Leaf extract from Skudai	67.41 ± 0.03^**^	64.76 ± 0.01^***^	55.77 ± 0.03^***^	50.44 ± 0.03^***^	49.61 ± 0.01^***^	47.07 ± 0.04^***^	8.42 ± 0.01^***^	11.65 ± 0.01^***^

Leaf extract from Batu Pahat	82.67 ± 0.07^*^	78.96 ± 0.02^**^	77.20 ± 0.02^*^	60.89 ± 0.01^***^	58.22 ± 0.01^***^	50.59 ± 0.01^***^	11.40 ± 0.01^***^	10.28 ± 0.01^***^

Leaf extract from Pulai	74.61 ± 0.08^**^	73.06 ± 0.07^**^	69.24 ± 0.09^*^	66.06 ± 0.10^**^	65.44 ± 0.10^*^	60.58 ± 0.07^*^	53.23 ± 0.03^**^	16.71 ± 0.02^***^

Values are mean ± STDEV for 3 replicates ^*^ *P* < 0.05, ^**^ *P* < 0.01, and ^***^ *P* < 0.001 compared to control (untreated cell).

**Table 3:** Percentage viability of MDA-MB-468 cells in leaf extracts from five different locations.

Concentration (*μ*g/mL)	7.81	15.63	31.25	62.5	125	250	500	1000
Leaf extract from Mersing	92.63 ± 0.04	76.53 ± 0.02^*^	14.21 ± 0.02^***^	7.13 ± 0.01^***^	3.78 ± 0.01^***^	3.63 ± 0.01^***^	3.38 ± 0.01^***^	6.91 ± 0.01^***^

Leaf extract from Muar	79.53 ± 0.03^**^	74.33 ± 0.02^***^	69.20 ± 0.09^*^	75.0 ± 0.03^**^	63.7 ± 0.01^*^	22.63 ± 0.02^***^	3.03 ± 0.01^***^	2.49 ± 0.01^***^

Leaf extract from Skudai	98.03 ± 0.082	101.67 ± 0.021	97.87 ± 0.016	82.9 ± 0.021^**^	77.0 ± 0.014^***^	52.33 ± 0.045^**^	4.83 ± 0.002^***^	4.43 ± 0.01^***^

Leaf extract from Batu Pahat	96.26 ± 0.02	92.06 ± 0.03^*^	91.23 ± 0.09	89.0 ± 0.04^*^	102.46 ± 0.06	64.61 ± 0.08^*^	7.07 ± 0.01^***^	6.19 ± 0.01^***^

Leaf extract from Pulai	92.96 ± 0.03	88.68 ± 0.03^*^	87.69 ± 0.02^**^	86.4 ± 0.01^**^	82.6 ± 0.04^***^	77.53 ± 0.04^**^	36.8 ± 0.07^**^	8.12 ± 0.01^***^

Values are mean ± STDEV for 3 replicates ^*^ *P* < 0.05, ^**^ *P* < 0.01, and ^***^ *P* < 0.001 compared to control (untreated cell).

**Table 4:** Percentage viability of MDA-MB-231 and MDA-MB-468 cells in kaempferol, naringenin, and a mixture of kaempferol and naringenin.

Cells	Concentration (*μ*g/mL)	3.91	7.81	15.63	31.25	62.5	125	250	500
MDA-MB-231	Kaempferol	82.16 ± 0.06	81.07 ± 0.06^***^	61.48 ± 1.59^**^	50.96 ± 0.09^***^	36.45 ± 0.04^*^	27.45 ± 0.06	43.52 ± 0.02^*^	77.37 ± 3.30^*^
	Naringenin	85.53 ± 0.06^*^	100.23 ± 0.06	87.13 ± 0.07	69.35 ± 0.02^***^	93.79 ± 0.07	83.97 ± 0.04^*^	47.91 ± 0.04^**^	13.48 ± 0.01^***^
	Mixture of kaempferol and naringenin	72.42 ± 0.11	66.94 ± 0.11^**^	62.17 ± 0.64^***^	56.10 ± 0.12^*^	27.15 ± 0.34^***^	26.37 ± 0.03^***^	23.42 ± 0.11^***^	23.32 ± 0.22^***^

MDA-MB-468	Kaempferol	96.00 ± 0.07	74.82 ± 0.02^**^	53.99 ± 0.03^***^	31.64 ± 0.03^***^	17.67 ± 0.02^***^	12.73 ± 0.01^***^	7.05 ± 0.01^***^	11.33 ± 0.03^***^
	Naringenin	85.62 ± 2.03	94.26 ± 2.30	117.71 ± 0.07^*^	112.18 ± 3.26	58.08 ± 1.51^*^	13.54 ± 0.02^***^	3.24 ± 0.01^***^	3.94 ± 0.01^***^
	Mixture of kaempferol and naringenin	92.02 ± 0.11	89.79 ± 0.01^***^	83.99 ± 0.98	70.82 ± 0.04^***^	29.38 ± 0.04^***^	16.44 ± 0.02^***^	13.34 ± 0.03^***^	13.33 ± 0.06^***^

Values are mean ± STDEV for 3 replicates ^*^ *P* < 0.05, ^**^ *P* < 0.01, and ^***^ *P* < 0.001 compared to control (untreated cell).
